# Effects of intranasal oxytocin on the self-perception and anxiety of singers during a simulated public singing performance: A randomized, placebo-controlled trial

**DOI:** 10.3389/fnins.2022.943578

**Published:** 2022-08-11

**Authors:** Flávia de Lima Osório, Gleidy Vannesa Espitia-Rojas, Lilian Neto Aguiar-Ricz

**Affiliations:** ^1^Ribeirão Preto Medical School, São Paulo University, Ribeirão Preto, SP, Brazil; ^2^National Institute of Science and Technology, Brasília, Brazil

**Keywords:** oxytocin, psychosocial stress, music performance anxiety, cognition, mood, singer, musicians

## Abstract

Professional musicians experience intense social exposure and high levels of preoccupation with their performance and potential negative reactions from the audience, which favor anxiety. Considering that oxytocin (OXT) has a potential therapeutic effect on anxiety, cognitive processes, and decreased psychosocial stress, this study’s objective was to assess the effects of a single dose of 24 UI of intranasal OXT among professional singers, during a public singing simulation test, on self-rated performance and mood. This crossover, randomized, double-blinded, placebo-controlled trial addressed 54 male singers with different levels of musical performance anxiety (42% high). The participants took part in different phases of a simulated public singing performance and completed instruments rating their performances (Self Statements During Public Performance- State version) and mood (Visual Analogue Mood Scale). Data were analyzed using ANOVA 2 × 2 for crossover trials. The results show that the use of OXT during the performance and immediate post-stress favored more positive (effect size: *d* > 1.04) and less negative assessments of musical performance (effect size: *d* > 1.86) than when placebo was used. No treatment effects were found in any VAMS subscales, indicating no direct anxiolytic effects. The conclusion is that OXT can minimizes social stress, especially during performances. This finding is exploratory and, if confirmed in future studies, may have relevance for musicians, especially those who constantly experience and recognize the impact of negative and catastrophic thoughts on performance and professional activities.

**Clinical Trial Registration:**

[https://ensaiosclinicos.gov.br/rg/RBR-5r5sc5], identifier [RBR-5r5sc5].

## Introduction

Being a professional musician necessarily involves intense social exposure inherent to the musical practice, which leads musicians to experience great apprehension regarding their performances and the possibility of negative audience responses. Hence, musicians often become anxious before a musical performance, and this anxiety is expressed in different forms and intensity, possibly favoring intense distress and/or compromising one’s response toward routine situations of the musical universe (Burin and Osório, 2017).

These experiences characterize what is called music performance anxiety (MPA), which can be understood as a subtype of social anxiety, as it is associated with a specific performance condition (Papageorgi et al., 2007; Taborsky, 2007; Ryan and Andrews, 2009; American Psychiatric Association [APA], 2013). A review study points out that the prevalence of MPA ranges from 15 to 25% worldwide (Steptoe, 2001).

Three groups of symptoms can characterize MPA, according to Valentine (2002) and Lehmann et al. (2007): physiological (for example: tachycardia, shortness of breath, hyperventilation, dry mouth, sweating, and muscle tension), mental (subdivided into cognitive - difficulty concentrating and memory, distorted and catastrophic thoughts; and emotional - stress, apprehension, insecurity, panic) and behavioral (agitation, technical failures, compromised performance, among others), which, in general, are experienced concomitantly (Steptoe, 2001; Marshall, 2008).

Catastrophic thoughts toward performance stand out among cognitive factors. Lehmann et al. (2007) call attention to the importance of cognitive symptoms in the maintenance of MPA and quality of performance. The presence of negative thoughts and lack of confidence regarding one’s musical performance appeared in an empirical study conducted by Burin et al. (2019) as common causes of anxiety, respectively reported by 47.4 and 39.2% of the musicians interviewed.

Singers stand out because their musical instrument is part of their bodies, so keeping an ideal physical and psychological condition is needed as the quality of one’s singing performance demands complete control of the singing voice, which is particularly sensitive to stress, putting even more pressure on singers, especially soloists (Ryan and Andrews, 2009; Larrouy-Maestri and Morsomme, 2014).

Some coping strategies are used to deal with MPA, such as those focused on emotional regulation. However, these are not always effective, and some situations require specialized treatment (Burin et al., 2019). A literature review (Burin and Osório, 2017) reports the efficiency of cognitive-behavioral therapies, whose primary purpose is cognitive restructuring, intended to alter thought patterns considered dysfunctional (Studer et al., 2011). Pharmacological treatments are seldom investigated or adopted, and the use of beta blockers and some anxiolytics is not feasible for musicians because it may compromise fine motor control and cognitive functions, impairing musical performance (Burin and Osório, 2016). Nonetheless, Nascimento (2013) found that 17.27% of undergraduate music students took beta-blockers without medical prescription to control their anxiety, especially when performing solo or participating in competitions or master classes. It can also be noted, among the coping strategies used by musicians, the use of alcohol and illicit substances as a way to manage symptoms of anxiety in musical performance (Ryan and Andrews, 2009).

Oxytocin (OXT) has shown a therapeutic potential associated with anxiety, cognitive processes, and decreased psychosocial stress (Heinrichs et al., 2003; Guastella et al., 2009a; Alvares et al., 2012; Oliveira et al., 2012; Harvey, 2020) among healthy individuals and those with pathologies.

The search for possible interactions/associations between singing and neurohormones such as OXT has been studied. Although the understanding of music neuroscience is far from being unanimous (Cohen and Neumann, 2022), some findings point to an increase in endogenous OXT levels after singing, especially in a group context (Grape et al., 2003; Keeler et al., 2015; Bowling et al., 2022).

Another study evaluated the effects of intranasal administration of OXT against MPA symptoms (Sabino et al., 2020). The findings of this clinical trial showing that OXT improved the recognition of joyful facial expression (social approval) in a computer task, an ability that was impaired among musicians with high levels of MPA. However, it did not show any effect on mood/anxiety indicators or cognition, which seemed to be associated with the experimental condition; i.e., it did not expose the musicians or represent a threat to the participants considering that OXT effects are context-dependent (Bartz et al., 2011). This study signaled the possibility of possible therapeutic effects of OXT for the management of MPA symptoms in musicians, which would need to be further explored.

Therefore, this study’s objective was to assess the effects of a single dose of intranasal OXT (24 IU) among professional singers during a simulated public singing test on self-rated performance (primary outcome) and mood (secondary outcome). The hypothesis is that OXT will improve the participants’ self-assessed performance and decrease anxiety symptoms without altering cognitive parameters, sedation, or discomfort measured in the VAMS, portraying a reduced side effect profile.

## Method

This randomized, double-blinded, placebo-controlled crossover trial was registered at the Brazilian Clinical Trials Registry, under No. RBR-5r5sc5 and approved by the Local Research Ethics Committee (process no. HCRP 4031/2015).

### Participants

A convenience sample included male professional, erudite or contemporary commercial music singers, aged 18 to 45, with experience/training in singing voice of at least 3 years and frequently performing in public (at least once a month). The participants were recruited in music schools, universities, and choirs in the city/region of Ribeirão Preto, SP, Brazil, and *via* the social media and local press. Exclusion criteria were: the presence of self-reported neurological or current or previous psychiatric disorders. Sampling calculation (G*Power 3.1.9.7 Software) estimated 50 participants, considering six measured points, a significance level of 0.05, power of 0.80 and minimum effect size of *f* = 0.15.

### Measurement instruments

The following instruments were used to characterize the study sample:

a. Kenny Music Performance Anxiety Inventory (KMPAI), proposed by Kenny et al. (2004), translated, adapted, and validated in Brazil by Barbar et al., 2014a,b. Musicians scoring ≥-15 are considered to have high MPA, while scores below this threshold indicate low MPA.
b. Self Statements During Public Performance (SSPS-P): a modified version of the Self Statements During Public Speaking scale developed by Hofmann and DiBartolo (2000). It was translated and adapted to Brazilian Portuguese by Osório et al. (2012). The instrument assesses self-perceptions regarding public performance based on cognitive theories of anxiety. It consists of 10 items divided into two subscales: positive self-assessment and negative self-assessment. The items are rated on a scale ranging from 0 (totally disagree) to 5 (totally agree). Higher scores indicate higher positive self-assessment, and lower scores indicate negative self-assessment (scores are inverted in the last subscale).
c. Self-Reporting Questionnaire (SRQ-20): instrument developed by Harding et al. (1980) to screen for mental disorders and validated for the Brazilian population by Mari and Williams (1986). The 20-item version was used, in which items are coded as dichotomous variables, with higher scores indicating higher levels of psychopathology.

The following instruments were used to assess the outcomes:

a. Self Statements During Public Performance (SSPS-P)- state version: the instrument reported in item b. The participants completed it considering the simulated public performance implemented in the experiment (Osório et al., 2013).
b. Visual Analogue Mood Scale (VAMS): translated and adapted to Brazilian Portuguese by Zuardi and Karniol (1981) and reviewed by Parente et al. (2005). The scale has 16 items, each formed by a pair of opposing adjectives connected by a 100-mm line. The subjects are asked to mark the line with a vertical trace, indicating how they feel according to each adjective. The center of the line corresponds to how the participant usually feels, and the two extremes indicate positive or negative increments in the intensity of the states described by the adjectives. The VAMS has four subscales: anxiety, sedation, cognitive impairment, and discomfort.

These scales have been widely adopted by experimental studies involving the Self Statements During Public Performance (SSPS-P) and are sensitive to the model (Bergamaschi et al., 2011; Oliveira et al., 2012; Osório et al., 2013; Linares et al., 2019).

### Procedure

The experimental procedure adopted was the Simulated Public Singing Test (SPST). The SPST was based on the Simulated Public Speaking experiment, initially proposed by McNair et al. (1982) and adapted to Brazil by Guimarães et al. (1987). Different variations of the Simulated Public Speaking test has been widely used (Osório et al., 2008) as an experimental model in different international clinical studies because of the significant results in inducing anxiety (Jezova et al., 2005; Bergamaschi et al., 2011; Zuardi et al., 2017; Angélico et al., 2018). It comprises six phases: initial measure, baseline, pre-stress/anticipation, execution/performance, and immediate and late post-stress/recovery. Physiological and subjective measurements are taken in each procedure phase (Osório et al., 2008). The SPST proposed here consists of an experimental situation in which singers are invited to participate in an anxiety test, the content of which is not revealed.

**FIGURE 1:**
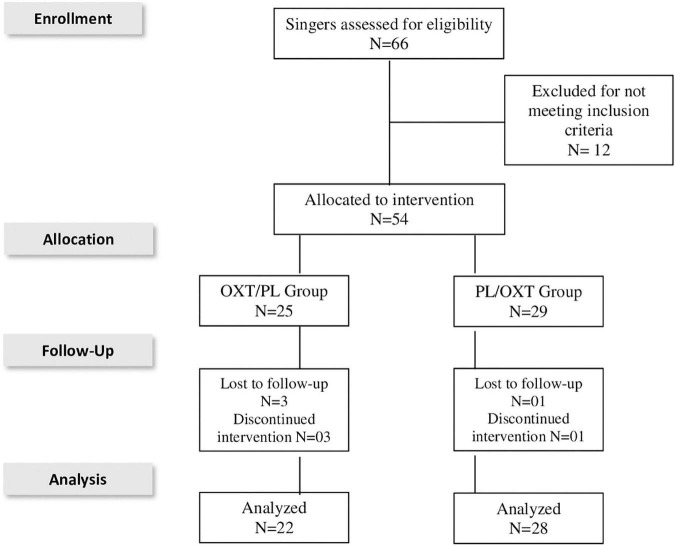
PRISMA flowchart — participants’ inclusion and exclusion.

The singers were randomized and assigned to two groups (simple randomization). One group received a single dose of intranasal OXT (24 IU), and the other group received a placebo (and vice-versa), according to recommendations proposed by Guastella et al. (2013). Both substances were packed in identical flasks and sequentially numbered according to the randomization list (computer-generated random numbers available at www.randomizacao.org.br), prepared by an investigator who was not involved in the trial. The evaluator (second author of the article) delivered the bottle to the participant and guided the application procedures. Both were blind to the substance administered. A dose of 24 IU was chosen based on evidence of positive effects from previous investigations and the absence of adverse effects (Bradley and Woolley, 2017; Leppanen et al., 2018; De Cagna et al., 2019; Sabino et al., 2020). Participation was voluntary, and all individuals signed free and informed consent forms. A total of 54 singers were assessed for eligibility and 50 singers concluded the study. Figure 1 presents the inclusion/exclusion process.

Data collection begins after a brief rapport and assessment of initial measures (IM), which involve instruments to characterize the sample and analyze the outcomes previously described. Next, each singer remains in the environment relaxing for approximately 15 min (magazines with neutral content are provided for distraction). Then, the experiment is initiated, and baseline measures (BM) related to the outcomes are taken. Next, the substance (OXT or placebo) is administered, and the singer is instructed to relax. After 35 min of waiting, the singer is invited to warm up his voice for 10 min, though warming up is optional. At the end of this period, instructions concerning the test itself are provided. The singers received the following instructions: “*You will perform a cappella interpretation of a song of your choice for four minutes. You will have two minutes to prepare. Your performance will be video and audio recorded and later assessed by a singing teacher and a speech therapist/voice coach.*”

Next, the outcome variables previously described (Pre-stress/Anticipation Measure – AM) are taken, and the singer is positioned in front of a microphone and a video camera, which transmits the performance image in real-time to a 24-inch monitor positioned in front of the singer. SoundForge 11.0 (Sony Creative Software Inc^®^., Middleton, WI, United States) in a sampling frequency of 44,100 Hz and 16 bits, in monophonic recording format and WAV storage was used for voice recording and later analysis of the vocal parameters (not presented in this study).

The musical performance is initiated, but it is interrupted at the end of the first 2 min, and the outcome measures are taken (Execution/Performance Measure – PM). Then, the performance is resumed to complete the 4 min, and a new measure of the outcome variables (Immediate Post-stress/Recovery Measure – Pe0M) is taken.

The singer remains in the place and is instructed to relax. After 20 min, the outcome variables are measured one last time (Late Post-stress/Recovery Measure – Pe1M), and the SPST is ended. Note that the singers are supposed to perform according to their musical styles in terms of posture, tuning, rhythm, meter, and vocal characteristics. “Supplementary Material” shows a summary of this experimental model.

Data were collected in a room where lighting, sound, and temperature were controlled. Singers were instructed not to eat or drink anything (except water) in the 2 h that preceded the session and not to exercise or consume alcohol or drinks containing xanthine 24 h before the experiment.

The second experimental session was scheduled between 15 and 30 days later to prevent memory effects (mean 19.4 ± 13.9 days). In this session, the participants were given the substance prescribed for their allocation group (participants treated with placebo in the first session received OXT in the second session and vice-versa) and completed the same steps of the previous session. The crossover design was chosen to minimize the influence of individual differences on the results.

### Data analyses

Descriptive statistics were used to analyze data in addition to comparison tests and analysis of variance (ANOVA 2 × 2) crossover models. The Omnibus test was also used as a measure of separability or independence between the treatment effect and carryover effect. Stata Statistical Software (StataCorp LP, 13.0 College Station, TX, United States) was used for the analyses based on the intention-to-treat principle. The effect size of the differences was calculated using Cohen’s *d* (Cohen, 1988).

## Results

The socio-demographic and clinical characterization of the sample is presented in Table 1.

**TABLE 1:** Sociodemographic and clinical characterization of the sample.

Variable	Total sample (*N* = 50)	OXT-PL (*N* = 22)	PL-OXT (*N* = 28)	Statistics
**Education N(%)**				
≤12 years	18 (36)	6 (27)	12 (43)	*p* = 0.25
>12 years	32 (64)	16 (73)	16 (57)	
**Music genre N(%)**				
Erudite	8 (16)	04 (18)	04 (14)	*p* = 0.70
Contemporary commercial music	42 (84)	18 (82)	24 (86)	
**Age –years $\bar{X}$ (SD)**	28.86 (6.08)	28.55 (6.15)	29.11 (6.14)	*p* = 0.77
**KMPAI $\bar{X}$ (SD)**	−23.04 (20.50)	−29.14 (21.46)	−18.25 (18.72)	*p* = 0.06
**KMPAI (score ≥-15) N(%)**	21 (42)	7 (32)	14 (50)	*p* = 0.75
**SSPS-P $\bar{X}$ (SD)**	4.62 (3.48)	5.09 (3.42)	4.25 (3.56)	*p* = 0.32
**SRQ-20 $\bar{X}$ (SD)**	4.62 (3.48)	5.09 (3.42)	4.25 (3.56)	*p* = 0.32

KMPAI, Kenny Music Performance Anxiety Inventory; SRQ-20, Self-Reporting Questionnaire; SSPS-P, Self Statements During Public Performance.

The participants were 28 years old on average, with high educational levels, and contemporary commercial music was the predominant musical style. The experimental groups did not differ in regard to any of the socio-demographic or clinical variables. Note that the medium scores related to MPA were above the cutoff point. According to the participants’ individual classification, 42% of them presented high levels MPA.

**FIGURE 2:**
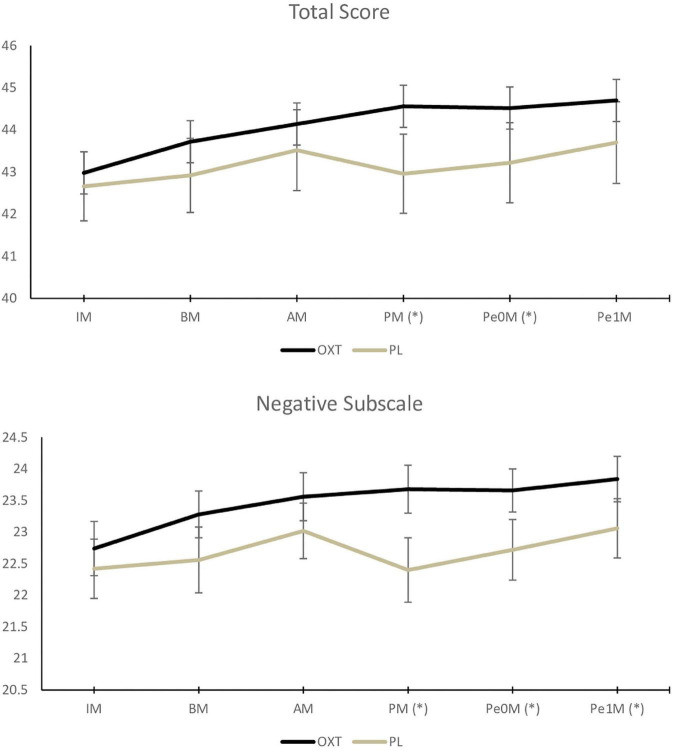
Effects of oxytocin and placebo on self-perception of performance (Self Statements During Public Performance–state version) (OXT, oxytocin; PLA, placebo; IM, Initial Measure; BM, Baseline Measure; AM, Pre-stress/Anticipation Measure; PM, Execution/Performance Measure; Pe0M, Immediate Post-stress/Recovery Measure; Pe1M, Late Post-stress/Recovery Measure; *difference with statistical significance = *p* < 0.05; error bar = standard deviation).

**FIGURE 3:**
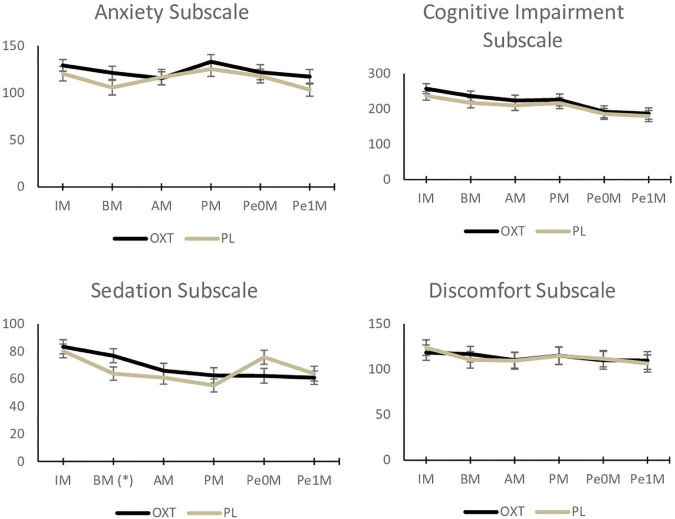
Effects of oxytocin and placebo on self-perception of performance (Visual Analogue Mood Scale) (OXT, oxytocin; PLA, placebo; IM, Initial Measure; BM, Baseline Measure; AM, Pre-stress/Anticipation Measure; PM, Execution/Performance Measure; PeOM, Immediate Post-stress/Recovery Measure; Pe1M, Late Post-stress/Recovery Measure; *difference with statistical significance = *p* < 0.05; error bar = standard deviation).

Figures 2, 3 and “Supplementary Material” present the study’s primary results.

The ANOVA results indicate an effect of the treatment in the participants’ self-perception during the performance and post-stress phases (especially: SSPS-P state version– total scale, performance phase: *F*(1,48) = 5.35, *p* = 0.03; SSPS-P state version– total scale, immediate post-stress phase: *F*(1,48) = 4.70, *p* = 0.04; SSPS-P state version– negative subscale, performance phase: *F*(1,48) = 8.69, *p* = 0.005; SSPS-P state version– negative subscale, immediate post-stress phase: *F*(1,48) = 7.04, *p* = 0.01; SSPS-P state version– negative subscale, late post-stress phase: *F*(1,48) = 6.06, *p* = 0.02. The singers assessed their performance more positively (effect size: *d* > 1.04) and less negatively (effect size: *d* > 1.86) when using OXT than when using placebo. The effects of the treatment were not found in any of the VAMS subscales, indicating a lack of anxiolytic effects (*p* > 0.06), or sedation-related potential side effects (*p* > 0.16 – change in sedation occurred before the substance was administered), cognitive impairment (*p* > 0.16), or discomfort (*p* > 0.42).

No effects were found related to the sequence or period of the experiment. The separability measure (Omnibus test) indicates that up to 29.3% of the estimated effects can be specifically attributed to the OXT effect.

## Discussion

This study was developed to assess the effects of a single dose of 24 UI of intranasal OXT among the singers’ self-perception of performance and mood. Considering that OXT effects are context-dependent, an experimental situation was adapted to simulate singing to an audience, and similarly to the simulated public speaking test, this experiment induced anxiety in a social threat context (Osório et al., 2008). Likewise, the scales used to assess performance and mood were sensitive to the experimental anxiety model as previously reported (Osório et al., 2008), with emphasis on the SSPS- P’s negative subscale.

Oxytocin improved the participants’ self-perception during and immediately after their performance, highlighting the effect of OXT in the cognitive component of MPA, with a high size effect. This finding is relevant given that negative and catastrophic thoughts related to MPA are common and favor harm and suffering, with loss of quality of life and work. Studies show that from 24 to 40% of musicians experience high levels of MPA (Barbar et al., 2014c; Burin et al., 2019), which, besides anxiety, include dysfunctional thoughts, often leading individuals to quit the profession when experiencing high levels of everyday stress (Barbar et al., 2014c). This population requires special care and support to cope with these experiences and often demands external help, while there are still limited therapeutic resources (Burin and Osório, 2016).

A previous study conducted with musicians in general (Sabino et al., 2020) reports that OXT did not present an effect on negative cognitions associated with self-perception but positively favored the recognition of facial emotions only among those with high levels of MPA.

Oxytocin’s favorable action in socially stressful situations was previously reported by studies addressing different samples. Guastella et al. (2009b) addressed individuals with social anxiety and reported that OXT associated with exposure therapy decreased negative mental representations after public speaking. In addition, an increase was found in the participants’ positive assessments of their appearance and speech performance, concluding that OXT facilitated a more adaptive and precise performance assessment. Quirin et al. (2011) applied the Trier Social Stress Test and verified that OXT decreased the release of cortisol among individuals with poor emotional regulation. The findings reported by Alvares et al. (2012) are also noteworthy as healthy men under the effect of OXT and facing an anxiety-inducing situation (speaking to a virtual audience) had negative beliefs toward their performance decreased and more frequently looked to faces with positive emotions and less frequently look to those with negative emotions.

Different from the expected, anxiety scores obtained throughout the SPST did not differ between treatments, indicating a lack of effect in the affective component of MPA, that is, a direct anxiolytic effect. However, note that the anxiety scores obtained between the baseline and pre-stress/anticipation decreased when OXT was used and increased under the placebo, suggesting a potential decrease in anticipation anxiety. This finding concerning OXT had been previously reported by Oliveira et al. (2012), who associated it with results found with diazepam in Zuardi et al. (1993); both studies simulated public speaking. On the other hand, Heinrichs et al. (2003) implemented a similar experimental situation (Trier Social Stress Test) and found that OXT improved calmness and decreased cortisol levels after stress, especially when associated with a social support condition, highlighting its anxiolytic effect.

These findings, taken together, suggest that OXT can minimize situations of social stress, either through direct effects on anxiety, social cognition, negative beliefs/perceptions or in physiological parameters such as cortisol, which can impact on social facilitation. Additionally, previous studies indicate the effects of OXT on self-confidence (Kosfeld et al., 2005), positive perception of personality traits (Cardoso et al., 2012), and self-view (Colonnello and Heinrichs, 2014), factors that can mediate and favorably modulate these social effects.

According to Guastella et al. (2008), OXT can also facilitate adaptive social learning, broadening the perception of positive social experiences, and decreasing the impact of experiences, often exaggerated, of social threat, especially among individuals with social anxiety symptoms, a condition similar to MPA (Kenny, 2011).

Different hypotheses possibly explain the OXT effects but are still uncertain. There is not enough consensus and evidence to conclude whether its actions are due to peripheral or central effects, and in the case of the latter, whether the concentrations that reach the brain are effective in reaching relevant areas, to the point of explaining the behavioral effects. Furthermore, it is still not possible to conclude whether its effects are primary or the result of an epiphenomenon, since OXT acts on many functions and behaviors (Leng et al., 2022). Although, according McCarthy and Altemus (1997) it is known that OXT influences many neurobehavioral functions and is distributed in the entire central nervous system (McCarthy and Altemus, 1997). Additionally, it presents an intracerebral inhibitory effect in the HPA axis when an individual faces a stress-induced activity, playing an essential role in its management (Neumann et al., 2000) and decreasing activation of the amygdala in the process of fear modulation and responsiveness to social stimuli (Huber et al., 2005; Kirsch et al., 2005; Domes et al., 2007).

Guastella et al. (2009a) propose an OXT bio-cognitive model, in which OXT improves the processing of positive social signs and decreases threat through top-down processes. In the same direction, Quirin et al. (2011) report that OXT stimulates prefrontal cortex activity, the brain area implicated in the regulation of emotional stress (Kalisch, 2009), and parasympathetic activity (Porges, 1995; Ahern et al., 2001). Alvares et al. (2012) also share the notion that OXT can improve the activation of regions such as the dorsal anterior cingulate cortex, left dorsolateral prefrontal cortex, and medial prefrontal cortex, which shows decreased functioning in situations of automatic attention to threatening stimuli (Bishop, 2007) and distorted negative beliefs, and can, when activated, favor cognitive reappraisal.

More recently, Thomas and Larkin (2020) assessed depressed patients and found that cortisol levels were positively correlated with the level of negative thoughts, while OXT showed an inverse correlation, suggesting that the intensity and frequency of negative thoughts mediate the relationship between stress and cortisol.

Given the previous discussion, the potential of OXT to minimize stress, especially in social situations, seem undeniable, and the results reinforce the interaction model proposed by Bartz et al. (2011) to explain the OXT effects. This model proposes that one or more basic mechanistic processes (e.g., decreased anxiety) are associated with contextual variables and individual differences that direct the effects.

As noted in study previously mentioned, negative cognitions were not verified in a non-socially threatening situation (Sabino et al., 2020). This study found these effects in a situation simulating threat and anxiety that musicians often experience. The individual characteristics of the sample were controlled only for sex. Unfortunately, due to sample power, previous levels of MPA could not be analyzed. Note, however, that the sample presented high levels of MPA; almost half of the sample presented levels above the instrument’s cutoff point.

Even though it was not this study’s objective, the use of OXT did not lead to cognitive impairment, sedation, or discomfort, as measured by the VAMS subscales. Additionally, the participants did not report any side effects, which is in line with the results reported by the meta-analysis conducted by MacDonald et al. (2011). Hence, this drug has some advantages over anxiolytics and beta-blockers, broadening the possibilities of use in performance situations such as those experienced by musicians, which demand motor coordination, attention, memory, and precision (Tubiana, 2001; Kenny et al., 2004). An assessment of the effect of OXT on acoustic parameters (e.g., fundamental frequency, jitter, and tremor) is being conducted, and preliminary results indicate no impact/impairment; thus, OXT is a very promising strategy for this target population.

This study’s limitations include a sample restricted to male participants and singers. Note that the objective quality of the performances during the use of substances was not assessed. Hence, we cannot affirm whether improved self-perception was in fact associated with a better performance, which should be further investigated in the future.

To date, most studies conducted with OXT have focused on acute use (Leng et al., 2022). Among studies with long-term use, although the evidence is favorable regarding symptoms of stress and anxiety and the side effect profile, it is exploratory (e. g., van Zuiden et al., 2017; Kou et al., 2022). Thus, in future studies, it seems opportune to investigate the effects associated with the chronic administration of OXT in musicians with high social performance anxiety, in order to try to establish its therapeutic potential, as already pointed out by Peters et al. (2014), Neumann and Slattery (2016), and Leng et al. (2022). Still, issues related to the therapeutic window, dose-dependence, among others, need to be explored in depth.

## Data availability statement

The raw data supporting the conclusions of this article will be made available by the authors, without undue reservation.

## Ethics statement

The studies involving human participants were reviewed and approved by Comitê de Ética em Pesquisa com Seres Humanos do Hospital das Clínicas da Faculdade de Medicina de Ribeirão Preto - USP. The patients/participants provided their written informed consent to participate in this study.

## Author contributions

FO, GE-R, and LA-R: conception and design, data collection, analysis, interpretation, and approval of the final version. FO: substantial contributions to the drafting or revising it critically for important intellectual content. All authors contributed to the article and approved the submitted version.
