# Supplementary material for: Effects of intranasal oxytocin on the self-perception and anxiety of singers during a simulated public singing performance: A randomized, placebo-controlled trial
Source: Front Neurosci. 2022 Aug 11;16:943578. doi: 10.3389/fnins.2022.943578 (PMC9403236; doi:10.3389/fnins.2022.943578)
Supplement: Supplementary file 1 [file Table_1.DOCX]

**Supplementary Material**

SM 1. Experimental model of Simulated Public Singing Performance

| Duration | Phase | Activities |
| --- | --- | --- |
| - 75 minutes | Initial  Measure (IM) | The participant arrives at the lab, rapport, completes characterization instruments (socio-demographic questionnaire, KMPAI, SSPS-P), assessment of outcome variables: *SSPS-P- state version and VAMS* |
| - 55 minutes | Baseline Measure (BM) | Experiment begins  Assessment of outcome variables: SSPS-P- state version and VAMS |
| - 50 minutes | Substance is administered (OXT or PL) | |
| - 15 minutes | Vocal warming up (optional) | |
| - 5 minutes | Pre-stress/Anticipation Measure (AM) | Assessment of outcome variables: *SSPS-P- state version and VAMS* |
| 0 | Musical Performance | |
| + 2 minutes | Execution/Performance (PM) | Musical performance is interrupted and outcome variables are assessed:  *SSPS-P- state version and VAMS* |
| + 4 minutes | Immediate Post-stress/Recovery Measure (Pe0M) | Musical performance ends  Assessment of outcome variables: *SSPS-P- state version and VAMS* |
| + 24 minutes | Late Post-stress/Recovery Measures (Pe1M) | Assessment of outcome variables: *SSPS-P- state version and VAMS*  Experiment is ended. |

SM2 – Mean scores of outcome measures, as a function of treatment over the different phases of the Public Singing Simulation Test

|  | **Initial** | | **Basal** | | **Anticipation** | | **Performance** | | **Post-stress Imediate** | | **Post-stress Late** | |
| --- | --- | --- | --- | --- | --- | --- | --- | --- | --- | --- | --- | --- |
|  | **Mean** | **(SD)** | **Mean** | **(SD)** | **Mean** | **(SD)** | **Mean** | **(SD)** | **Mean** | **(SD)** | **Mean** | **(SD)** |
| SSPS-P- state version - Total Score | | | | | | | | | | | | |
| **OXT** | 42.98 | 0.82 | 43.72 | 0.85 | 44.14 | 0.88 | 44.56 | 0.95 | 44.52 | 0.92 | 44.7 | 0.95 |
| **PL** | 42.66 | 0.82 | 42.92 | 0.88 | 43.52 | 0.96 | 42.96 | 0.94 | 43.22 | 0.95 | 43.7 | 0.97 |
| **SSPS-P- state version - Negative Subscale** | | | | | | | | | | | | |
| **OXT** | 22.74 | 0.43 | 23.28 | 0.37 | 23.56 | 0.38 | 23.68 | 0.38 | 23.66 | 0.34 | 23.84 | 0.36 |
| **PL** | 22.42 | 0.47 | 22.56 | 0.52 | 23.02 | 0.44 | 22.40 | 0.51 | 22.72 | 0.48 | 23.06 | 0.47 |
| **VAMS – Anxiety Subscale** | | | | | | | | | | | | |
| **OXT** | 129.18 | 6.18 | 121.22 | 7.02 | 115.42 | 6.94 | 132.96 | 7.62 | 121.86 | 8.00 | 117.10 | 7.73 |
| **PL** | 120.32 | 7.78 | 105.44 | 7.8 | 116.62 | 8.28 | 125.22 | 7.81 | 117.8 | 7.31 | 103.34 | 6.95 |
| **VAMS – Cognitive Impairment Subscale** | | | | | | | | | | | | |
| **OXT** | 257.36 | 14.17 | 236.46 | 14.38 | 223.98 | 15.15 | 225.84 | 16.65 | 191.96 | 17.08 | 187.06 | 16.10 |
| **PL** | 237.30 | 12.01 | 217.24 | 14.17 | 209.80 | 14.07 | 216.70 | 15.67 | 186.29 | 15.54 | 179.92 | 15.63 |
| **VAMS – Sedation Subscale** | | | | | | | | | | | | |
| **OXT** | 83.34 | 5.11 | 76.82 | 5.10 | 66.0 | 5.34 | 62.50 | 5.63 | 62.30 | 5.37 | 60.84 | 4.89 |
| **PL** | 80.32 | 4.97 | 63.82 | 4.82 | 60.96 | 4.80 | 55.24 | 4.72 | 75.72 | 5.06 | 63.82 | 5.39 |
| **VAMS – Discomfort Subscale** | | | | | | | | | | | | |
| **OXT** | 118.32 | 8.42 | 116.72 | 8.59 | 110.14 | 8.86 | 115.00 | 9.56 | 109.90 | 9.73 | 109.74 | 9.74 |
| **PL** | 123.82 | 8.56 | 110.46 | 9.15 | 109.38 | 9.07 | 114.92 | 9.65 | 111.62 | 9.03 | 106.42 | 9.42 |

AM: Pre-stress/Anticipation Measure; BM: Basal Measure; IM: Initial Measure; PM: Execution/Performance Measure; Pe0M: Post-stress/Recovery Imediate Measure; Pe1M: Post-stress/Recovery Late Measure

SM3 – Effects of oxytocin on performance self-assessment and mood indicators, as a function of the different phases of the Public Singing Simulation Test

|  | ***Intersubjects*** | | | | ***Intrasubjects*** | | | |
| --- | --- | --- | --- | --- | --- | --- | --- | --- |
|  | ***Sequence effect*** | | ***Residual/Carryover*** | | ***Treatment effect*** | | ***Period effect*** | |
| ***Variable*** | ***F(1,48)*** | ***p-value*** | ***F(1,48)*** | ***p-value*** | ***F(1,48)*** | ***p-value*** | ***F(1,48)*** | ***p-value*** |
| ***SSPS-P- state version - Total Score*** | | | | | | | | |
| Initial | 0.53 | 0.48 | 0.52 | <0.001* | 0.13 | 0.72 | 1.73 | 0.20 |
| Basal | 0.07 | 0.79 | 6.90 | <0.001* | 1.50 | 0.22 | 0.18 | 0.67 |
| Anticipation | 0.08 | 0.78 | 9.95 | <0.001* | 1.17 | 0.29 | 0.01 | 0.91 |
| Performance | 0.02 | 0.90 | 6.83 | <0.001* | 5.35 | 0.03* | 0.00 | 0.94 |
| Post-stress Imediate | 0.06 | 0.80 | 8.86 | <0.001* | 4.70 | 0.04* | 0.03 | 0.87 |
| Post-stress Late | 0.04 | 0.83 | 11.32 | <0.001* | 2.88 | 0.10 | 0.64 | 0.43 |
| ***SSPS-P- state version - Negative Subscale*** | | | | | | | | |
| Initial | 0.01 | 0.09 | 6.55 | <0.001* | 0.29 | 0.59 | 1.73 | 0.20 |
| Basal | 0.03 | 0.86 | 5.41 | <0.001* | 3.31 | 0.08 | 0.18 | 0.67 |
| Anticipation | 0.58 | 0.45 | 4.65 | <0.001* | 2.09 | 0.15 | 0.01 | 0.91 |
| Performance | 0.22 | 0.64 | 3.43 | <0.001* | 8.69 | 0.005* | 0.00 | 0.94 |
| Post-stress Imediate | 0.16 | 0.69 | 4.79 | <0.001* | 7.04 | 0.01* | 0.03 | 0.87 |
| Post-stress Late | 0.33 | 0.57 | 6.58 | <0.001* | 6.06 | 0.02* | 0.64 | 0.43 |
| **VAMS – Anxiety Subscale** | | | | | | | | |
| Initial | 0.64 | 0.43 | 1.86 | 0.02* | 1.65 | 0.21 | 3.35 | 0.08 |
| Basal | 0.36 | 0.55 | 2.10 | <0.001* | 3.60 | 0.06 | 0.18 | 0.67 |
| Anticipation | 2.30 | 0.14 | 1.79 | 0.03* | 0.00 | 0.96 | 0.46 | 0.50 |
| Performance | 0.65 | 0.42 | 1.73 | 0.03* | 1.21 | 0.28 | 4.98 | 0.03* |
| Post-stress Imediate | 0.33 | 0.57 | 2.54 | <0.001* | 0.32 | 0.58 | 0.35 | 0.55 |
| Post-stress Late | 0.00 | 0.95 | 2.16 | 0.004* | 2.70 | 0.11 | 0.00 | 0.94 |
| **VAMS – Cognitive Impairment Subscale** | | | | | | | | |
| Initial | 0.53 | 0.47 | 3.40 | <0.001* | 2.45 | 0.12 | 0.02 | 0.89 |
| Basal | 1.10 | 0.30 | 4.13 | <0.001* | 2.00 | 0.16 | 0.68 | 0.41 |
| Anticipation | 1.42 | 0.24 | 5.42 | <0.001* | 1.55 | 0.22 | 0.04 | 0.84 |
| Performance | 1.69 | 0.20 | 6.83 | <0.001* | 0.41 | 0.52 | 1.54 | 0.22 |
| Post-stress Imediate | 0.83 | 0.37 | 5.41 | <0.001* | 0.26 | 0.61 | 1.87 | 0.18 |
| Post-stress Late | 2.83 | 0.10 | 5.51 | <0.001* | 0.35 | 0.56 | 0.30 | 0.58 |
| **VAMS – Sedation Subscale** | | | | | | | | |
| Initial | 0.93 | 0.34 | 3.09 | <0.001* | 0.50 | 0.48 | 0.81 | 0.37 |
| Basal | 0.27 | 0.61 | 3.36 | <0.001* | 7.18 | 0.01* | 0.01 | 0.91 |
| Anticipation | 0.85 | 0.36 | 4.78 | <0.001* | 1.18 | 0.28 | 0.65 | 0.42 |
| Performance | 2.95 | 0.09 | 4.66 | <0.001* | 2.02 | 0.16 | 5.04 | 0.03* |
| Post-stress Imediate | 1.94 | 0.17 | 4.57 | <0.001* | 0.83 | 0.37 | 1.48 | 0.23 |
| Post-stress Late | 3.23 | 0.08 | 6.57 | <0.001* | 0.58 | 0.45 | 0.15 | 0.70 |
| **VAMS – Discomfort Subscale** | | | | | | | | |
| Initial | 1.05 | 0.31 | 3.82 | <0.001* | 0.67 | 0.42 | 0.89 | 0.35 |
| Basal | 0.61 | 0.44 | 3.46 | <0.001* | 0.28 | 0.60 | 3.26 | 0.08 |
| Anticipation | 2.65 | 0.11 | 3.50 | <0.001* | 0.00 | 0.97 | 3.50 | 0.28 |
| Performance | 1.74 | 0.19 | 4.09 | <0.001* | 0.25 | 0.88 | 5.72 | 0.18 |
| Post-stress Imediate | 2.18 | 0.19 | 4.71 | <0.001* | 0.04 | 0.62 | 3.02 | 0.02* |
| Post-stress Late | 2.18 | 0.15 | 4.71 | <0.001* | 0.04 | 0.83 | 3.02 | 0.09 |
